# Functional and Radiological Outcome of Unstable Distal End Radius Fractures After Management With Closed Reduction and Fixation With Percutaneous Pinning Versus Closed Reduction and Colles’ Cast Application: A Comparative Study

**DOI:** 10.7759/cureus.97725

**Published:** 2025-11-25

**Authors:** Nischay Kaushik, Vedant Bajaj, Muhammed N Mancheri, Sharandeep S Saluja, Salman Durrani, Vaibhav Sanchay

**Affiliations:** 1 Orthopaedics, Dr. Baba Saheb Ambedkar Medical College and Hospital, New Delhi, IND; 2 Orthopaedics, IQRAA International Hospital and Research Centre, Kozhikode, IND

**Keywords:** closed reduction, colles’ cast, distal radius fracture, functional outcomes, percutaneous pinning, radiological outcome

## Abstract

Background: Distal end radius fractures are a common orthopaedic presentation and among the most frequent fractures in adults. Management options include closed reduction with Colles' cast immobilisation (CRCI) and closed reduction with percutaneous pinning (CRPP). This study aimed to evaluate and compare the functional and radiological outcomes of two treatment modalities for unstable distal radius fractures.

Methods: A prospective comparative study was conducted in the Department of Orthopaedics of Dr. Baba Saheb Ambedkar Medical College and Hospital, a tertiary care centre in New Delhi, India, spanning 18 months from July 2022 to July 2025. This study was conducted on 80 patients with unstable distal end radius fractures, allocated alternately into two groups: CRCI and CRPP. Patients were assessed clinically and radiologically at regular intervals. Functional evaluation was carried out using the Mayo Wrist Score, Modified Gartland and Werley Score, and Green and O’Brien Scoring System. Radiographic assessment followed Sarmiento’s modification of Lindstrom’s criteria. Data analysis was performed using IBM SPSS Statistics for Windows, Version 20 (IBM Corp., Armonk, NY, USA), with a *p*-value of <0.05 considered statistically significant.

Results: The CRPP group showed significantly better radiological parameters, including radial length, inclination, and volar tilt, compared to the CRCI group (*p* < 0.05). The CRPP group demonstrated better functional outcomes, with significantly higher Mayo Wrist, Modified Gartland and Werley, and Green and O’Brien Scores. Moreover, forearm rotation (supination and pronation) and wrist motion (radial and ulnar deviation) were markedly improved in the CRPP group.

Conclusions: CRPP demonstrated superior functional and radiological outcomes compared to Colles’ cast immobilisation for unstable distal end radius fractures. This technique allows more precise fracture reduction under fluoroscopic guidance and is recommended as the preferred option in suitable patients.

## Introduction

The distal radius, with its complex anatomy including the radial styloid, carpal articular surfaces, and Lister’s tubercle, is prone to a wide spectrum of fractures. These injuries range from simple extra-articular to complex intra-articular fractures and account for nearly 17.5% of all adult fractures presenting to emergency departments [1-3].

These fractures display a bimodal distribution, typically resulting from high-energy injuries in younger adults and low-energy falls in elderly women with osteoporosis [4]. Several classification systems, including those by Gartland and Werley and Frykman, have been proposed to describe these injuries, focusing on fracture location, articular involvement, and displacement [5,6]. Unstable fractures are characterized by criteria such as dorsal comminution, involvement of the volar buttress, fracture angulation >20°, radial shortening >5 mm, intra-articular step-off >2 mm, distal radioulnar joint incongruity, or failed conservative treatment [7,8].

Management strategies have shifted from conservative casting to surgical fixation, particularly in young or active patients with unstable displaced fractures. Percutaneous Kirschner wire pinning remains a minimally invasive, cost-effective option that provides stability while preserving soft tissue integrity and enabling early mobilisation [7,8]. By contrast, closed reduction and Colles’ cast immobilisation (CRCI) are still widely used for patients with low functional demands and stable fracture patterns [4,5]. The present study was designed to compare the functional and radiological outcomes of unstable distal radius fractures managed by two different treatment modalities: closed reduction with Colles’ cast immobilisation and closed reduction with percutaneous Kirschner wire (K-wire) fixation. The primary objective was to evaluate and compare the clinical and radiological results of these two techniques. In addition, the study aimed to assess the effectiveness of each method and to record any treatment-related complications.

## Materials and methods

A prospective comparative study was conducted in the Department of Orthopaedics of Dr. Baba Saheb Ambedkar Medical College and Hospital, a tertiary care centre in New Delhi, India, spanning 18 months from July 2022 to July 2025. The study was initiated after taking provisional acceptance (April 2022) for the protocol from the Institutional Research Ethics Board. However, the final acceptance (IEC No.: F.4(86)/2022/BSAH/DER) for the protocol was received in April 2023. A total of 80 patients with unstable distal radius fractures were included and alternately allocated into two groups: the closed reduction with percutaneous pinning (CRPP) group (n = 40), managed with closed reduction and percutaneous Kirschner wire fixation, and the CRCI group (n = 40), treated with CRCI.

Inclusion criteria comprised closed distal radius fractures fulfilling instability criteria, patients aged 18 years or older, and fresh injuries. Exclusion criteria comprised patients under 18 years of age, those with pre-existing wrist pathology or malunited distal radius fractures, pathological fractures, ipsilateral upper-limb fractures, open fractures, non-unions, or chronic injuries, as well as patients who were lost to follow-up or had less than six months of follow-up. Eligible participants were recruited from the Emergency Department and Orthopaedic outpatient clinics. All patients underwent initial evaluation with clinical assessment and laboratory investigations, including complete blood count, renal function tests, serum electrolytes, and viral markers. Chest radiography was performed in operative candidates, and dual-energy X-ray absorptiometry (DEXA) scans were obtained in elderly patients.

Interventions

**Figure 1:**
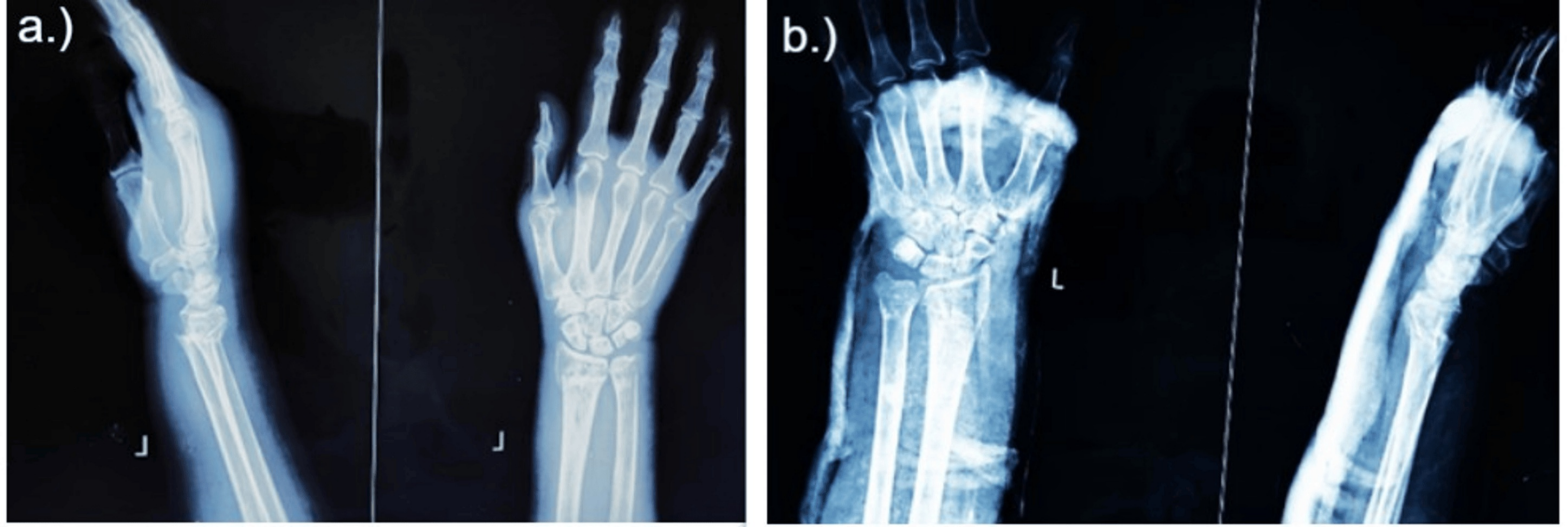
X-ray of the wrist: anteroposterior view and lateral view. A) Distal end radius fracture. B) After close reduction and Colle’s cast application.

Closed reduction and Colles cast application were performed under regional anaesthesia using a three-step manoeuvre: traction, correction of lateral displacement, and correction of dorsal displacement. After reduction, an above-elbow Colles cast was applied with proper moulding (Figure 1).

**Figure 2:**
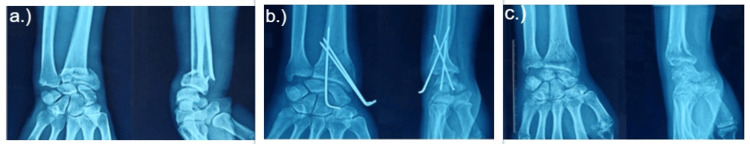
X-ray of the wrist: Anteroposterior view and lateral view. A) Distal end radius fracture. B) Closed reduction and percutaneous pinning of distal end radius fracture. C) Post-operative status healed distal end radius fracture after pin removal.

Closed reduction and percutaneous pinning was performed under regional/general anaesthesia under C-arm fluoroscopic guidance. Following reduction, Kirschner wires were inserted percutaneously. Typically, one or two wires were passed across the radial styloid, another from Lister’s tubercle to the volar cortex, and, when required, additional transulnar wires were used for distal radioulnar joint stabilization. Wire placement was confirmed under fluoroscopy (Figure 2).

Post-procedure, analgesia was provided, radiographs were checked, and patients were instructed to maintain limb elevation in a sling and perform active finger exercises. Patients were reviewed at two, three, and six weeks, then every six weeks, and at three-month intervals. Casts and Kirschner wires were removed after six weeks. Functional outcomes were assessed using Mayo Wrist Score [9], Modified Gartland and Werley Score [5], and Green and O’Brien Score [10]. Radiological outcomes were evaluated according to Sarmiento’s modification of Lindstrom’s criteria [11]. The scoring systems for the wrist function and radiological assessment is shown in the Appendix.

 Data analysis was performed using IBM SPSS Statistics for Windows, Version 20.0 (released 2011, IBM Corp., Armonk, NY). Continuous variables were reported as mean ± SD or median (IQR), and categorical variables as frequencies and percentages. Comparisons between groups were conducted using Student’s t-test, chi-square test, Fisher’s exact test, or Mann-Whitney U test as appropriate. A p-value <0.05 was considered statistically significant.

## Results

At the final follow-up, palmar flexion (68.8 ± 2.51° vs. 67.0 ± 2.05°; p = 0.038) was significantly better in the CRPP group, while dorsal flexion showed no significant difference (p = 0.30) (Table 1). Radial and ulnar deviation were both superior in CRPP (21.2 ± 4.27° vs. 16.25 ± 1.75°, p = 0.0001; 28.8 ± 3.4° vs. 25.6 ± 3.22°, p = 0.004) (Table 2). Supination (74.8 ± 5.4° vs. 68.3 ± 4.36°, p = 0.001) and pronation (65.05 ± 2.2° vs. 61.7 ± 3.1°, p = 0.001) were also significantly greater in the CRPP group (Table 3).

**Table 1:** Comparison of patients according to various movements of the wrist in the CRPP and CRCI groups. Values are expressed as mean ± SD. Statistical tests applied as appropriate (Independent Student’s t-test); p < 0.05 considered significant. CRCI: closed reduction with Colles' cast immobilisation, CRPP: closed reduction with percutaneous pinning

Time interval	Management	Number	Mean ± SD	p- value	t-value
According to the palmer flexion movement of the wrist (in degrees)					
At 6 weeks	CRPP	40	61.7 ± 3.6	0.07	2.11
	CRCI	40	60.2 ± 3.7		
At 12 weeks	CRPP	40	66.0 ± 2.58	0.3	1.1
	CRCI	40	65.2 ± 3.74		
At 24 weeks	CRPP	40	68.5 ± 2.81	0.045	2.05
	CRCI	40	67.00 ± 3.05		
At the final follow-up	CRPP	40	68.8± 2.51	0.038	3.51
	CRCI	40	67.00 ± 2.05		
According to the dorsal flexion movement of the wrist (in degrees)					
At 6 weeks	CRPP	40	60.5 ± 3.8	0.12	1.5
	CRCI	40	59.2 ± 3.3		
At 12 weeks	CRPP	40	63.5 ± 2.32	0.35	0.96
	CRCI	40	63.0 ± 2.4		
At 24 weeks	CRPP	40	64.5 ± 1.51	0.4	0.61
	CRCI	40	64.25 ± 1.85		
At the final follow-up	CRPP	40	67.5 ± 1.56	0.3	1.63
	CRCI	40	66.24 ± 1.8		

**Table 2:** Comparison of patients according to the radial and ulnar deviation movements of the wrist in the CRPP and CRCI groups. Values are expressed as mean ± SD for continuous variable. Statistical tests applied as appropriate (independent Student’s t-test); p < 0.05 considered significant. CRCI: closed reduction with Colles' cast immobilisation, CRPP: closed reduction with percutaneous pinning

Time interval	Management	Number	Mean ± SD	p- value	t- value
According to the radial deviation (in degrees)					
At 6 weeks	CRPP	40	14 ± 3.4	0.01	2.67
	CRCI	40	12.2 ± 2.5		
At 12 weeks	CRPP	40	18.5 ± 3.92	0.001	4.34
	CRCI	40	15.0 ± 2.26		
At 24 weeks	CRPP	40	20.2 ± 4.37	0.001	6.51
	CRCI	40	15.25 ± 1.95		
At the final follow-up	CRPP	40	21.2 ± 4.27	0.001	6.51
	CRCI	40	16.25 ± 1.75		
According to the ulnar deviation (in degrees)					
At 6 weeks	CRPP	40	22.5 ± 3.4	0.01	4.05
	CRCI	40	19.5 ± 3.5		
At 12 weeks	CRPP	40	26.5 ± 3.92	0.001	4.86
	CRCI	40	23.0 ± 3.26		
At 24 weeks	CRPP	40	26.7 ± 3.37	0.004	3.31
	CRCI	40	24.5 ± 3.16		
At the final follow-up	CRPP	40	28.8 ± 3.4	0.004	4.38
	CRCI	40	25.6 ± 3.22		

**Table 3:** Comparison of patients according to the supination and pronation movements of the wrist in the CRPP and CRCI groups. Values are expressed as mean ± SD for continuous variables. Statistical tests applied as appropriate (independent Student’s t-test); p < 0.05 considered significant. CRCI: closed reduction with Colles' cast immobilisation, CRPP: closed reduction with percutaneous pinning

Time interval	Management	Number	Mean ± SD (in degrees)	P -value	t-value
According to the supination movement					
At 6 weeks	CRPP	40	66.9 ± 5.78	0.001	3.82
	CRCI	40	62.2 ± 4.9		
At 12 weeks	CRPP	40	71.5 ± 6.22	0.001	3.96
	CRCI	40	66.2 ± 5.02		
At 24 weeks	CRPP	40	73.5 ± 5.7	0.001	4.69
	CRCI	40	67.5 ± 4.96		
At the final follow-up	CRPP	40	74.8 ± 5.4	0.001	5.98
	CRCI	40	68.3 ± 4.36		
According to the pronation movement					
Time interval	Management	Number	Mean ± SD (in degrees)	p- value	t-value
At 6 weeks	CRPP	40	59.0 ± 4.3	0.001	4.25
	CRCI	40	55.0 ± 3.9		
At 12 weeks	CRPP	40	63.9 ± 2.77	0.001	5.26
	CRCI	40	59.7 ± 2.92		
At 24 weeks	CRPP	40	64.15 ± 2.48	0.001	5
	CRCI	40	61.0 ± 3.4		
At the final follow-up	CRPP	40	65.05 ± 2.2	0.001	5.92
	CRCI	40	61.7 ± 3.1		

By the Mayo Wrist Score, 67.5% of the CRCI patients had only fair outcomes, compared with 47.5% of the CRPP patients who achieved good results (p = 0.007). Similar trends were observed in the Modified Gartland and Werley Score (p = 0.004), Green and O’Brien Score (p = 0.007), and Sarmiento’s criteria (p = 0.004), all favoring CRPP (Table 4).

**Table 4:** Comparison of the postoperative functional outcome in the CRPP and CRCI groups based on various scoring systems. Values are expressed as mean ± SD for continuous variables and N (%) for categorical variables. Statistical tests applied as appropriate (Chi-square test); p < 0.05 considered significant. CRCI: closed reduction with Colles' cast immobilisation, CRPP: closed reduction with percutaneous pinning

Result	Excellent, N (%)	Good , N (%)	Fair , N (%)	Poor , N (%)	p- value	χ²-value
Mayo Wrist Score						
CRPP	8 (20)	19 (47.5)	12 (30)	1 (2.5)	0.007	7.29
CRCI	3 (7.5)	7 (17.5)	27 (67.5)	3 (7.5)		
Modified Gartland and Werley demerit scoring system						
CRPP	6 (15)	18 (45)	14 (35)	2 (5)	0.004	8.21
CRCI	2 (5)	6 (15)	29 (72.5)	3 (7.5)		
Green and O’Brien Score (Cooney Modification)						
CRPP	8 (20)	19 (47.5)	12 (30)	1 (2.5)	0.007	7.29
CRCI	3 (7.5)	7 (17.5)	27 (67.5)	3 (7.5)		
Sarmiento’s Modification of Lindstorm Criteria						
CRPP	7 (17.5)	18 (45)	13 (32.5)	2 (5)	0.004	8.21
CRCI	4 (10)	9 (22.5)	24 (60.0)	3 (7.5)		

## Discussion

Distal radius fractures represent a significant portion of orthopaedic injuries in the upper limb and are a common presentation in the emergency department, comprising approximately 17.5% of all adult bone injuries. These fractures are more prevalent as individuals age and are associated with risk factors related to osteoporosis. Notably, distal radius fractures exhibit a bimodal age distribution, with an increased incidence among the elderly population. Factors such as reduced bone mineral density, White ethnicity, female gender, early menopause, and a family history of fractures contribute to the elevated risk of these injuries in older individuals. A common mechanism of injury for distal radius fractures is a fall onto an outstretched hand with the wrist in dorsiflexion, typically resulting in a Colles’ fracture. The demographic profile in our study is consistent with previous reports, showing a mean age of 52.83 ± 10.83 years in the CRPP group and 56.03 ± 14.18 years in the CRCI group, with no statistically significant difference. Similar to earlier studies, the 50-70 years' age range was the most frequently affected, representing 50.0% of patients in the CRPP group and 67.5% in the CRCI group. In our study, gender distribution showed that females were more frequently affected by distal radius fractures, comprising 55.0% in the CRPP group and 72.5% in the CRCI group. This observation aligns with previous studies by Knirk JL et al. [12], Orbay JL et al. [13], Mah ET et al. [2], and Vasudevan P et al. [14], which reported that 60-87% of patients were female. Regarding laterality, our study demonstrated a predominance of right-hand involvement, with 70% in the CRPP group and 75% in the CRCI group. This finding is consistent with prior research by Anakwe RE et al. [15], Orbay JL et al. [13], Knirk JL et al. [12], and Shrestha B et al. [16], who reported right-hand involvement in 55-77% of cases. The preference for the dominant hand is likely attributable to individuals instinctively using it to break or mitigate a fall.

The primary mode of injury in our study, as in previous research, was a fall onto an outstretched hand, which accounted for 90% of cases in the CRCI group and 82.5% in the CRPP group. This mechanism of injury is consistently identified as the most common cause of distal radius fractures in the literature, with studies by Clancey GJ et al. [17], Farhang Tajeddin et al. [18], Shrestha B et al. [16], and Anakwe RE et al. [15] reporting that 72-92% of patients sustained fractures due to falls on an outstretched hand. Our study further categorised patients with distal radius fractures according to Frykman's classification, revealing that Frykman type VI fractures were the most common in both the CRPP (37.5%) and CRCI (32.5%) groups. This finding is consistent with previous research by Clancey GJ et al. [17], Tajeddin F et al. [18], Shrestha B et al. [16], and Anakwe RE et al. [15], who also reported Frykman type VI as the most common fracture pattern. In our study, functional outcomes and radiographic parameters were evaluated in both the CRPP and CRCI groups at the final follow-up. CRPP demonstrated superior results compared to CRCI in terms of radial length, radial inclination, volar tilt, palmar flexion, dorsal flexion, supination, pronation, radial deviation, and ulnar deviation. These findings are consistent with previous studies by Knirk JL et al. [12], Mah et al. [2], Anakwe RE et al. [15], and Fitoussi F et al. [19], which reported similar ranges of motion and radiographic outcomes.

This study has several limitations. The relatively small sample size, single-centre design, and short follow-up period may limit the generalizability of our findings. The lack of randomisation and blinding could introduce selection and observer bias. Furthermore, long-term complications and functional recovery beyond the follow-up period were not assessed.

## Conclusions

Our study provides valuable insights into distal radius fractures and their management. Distal radius fractures are more common in older females. CRPP result in better functional outcomes compared to CRCI. Patients treated with CRPP exhibit a wider range of wrist movements and allow for more precise reduction under C-arm guidance as compared to those treated with closed reduction and cast immobilisation. Based on our findings, we recommend CRPP as the preferred treatment approach for unstable fractures of the distal end of the radius.

## Appendices

**Table 5:** Scoring Systems for Wrist Function and Radiological Assessment

Scoring System	Parameters	Scoring Details	Total Points	Result Classification
Green and O’Brien Score [10]	Pain	25 = none, 20 = mild occasional, 15 = moderate tolerable, 0 = severe intolerable	25	
	Range of Motion	100% = 25, 75-99% = 15, 50-74% = 10, 25-49% = 5, 0-24% = 0	25	
	Grip Strength	100% = 25, 75-99% = 15, 50-74% = 10, 25-49% = 5, 0-24% = 0	25	
	Activities (work/daily activity)	25 = regular employment, 20 = restricted employment, 15 = able to work but unemployed, 0 = unable to work due to pain	25	
	Total		100	Excellent: 90-100; Good: 80-89; Fair: 65-79; Poor: <65
Gartland and Werley Score [5]	Subjective Evaluation	Excellent (0), Good (2), Fair (4), Poor (6)	0-6	
	Objective Evaluation (loss of movement points)	Dorsiflexion (5), ulnar deviation (3), supination (2), palmar flexion (1), radial deviation (1), circumduction (1), distal radioulnar joint restriction (1)	Variable	
	Residual Deformity	Prominent ulnar styloid (1), residual dorsal tilt (2), radial deviation (2-3)	Variable	
	Complications	Arthritic changes (1-5), nerve complications (1-3), finger function (1-2)	Variable	
	Total	Sum of all above	Variable	Excellent: 0-2; Good: 3-8; Fair: 9-20; Poor: ≥21
Sarmiento Radiological Score [11]	Deformity severity	Based on dorsal angulation, shortening, and loss of radial deviation		Excellent: no/insignificant deformity; Good: slight deformity; Fair: moderate deformity; Poor: severe deformity
	Dorsal angulation	Excellent ≥ 0°, Good 1-10°, Fair 11-14°, Poor >15°		
	Shortening	Excellent <3 mm, Good 3-6 mm, Fair 7-11 mm, Poor >12 mm		
	Loss of radial deviation	Excellent <4°, Good 5-9°, Fair 10-14°, Poor >15°		
Mayo Wrist Score [9]	Pain	25 = none, 20 = mild occasional, 15 = moderate tolerable, 0 = severe intolerable	25	
	Functional Status	25 = regular employment, 20 = restricted employment, 15 = able to work but unemployed, 0 = unable to work due to pain	25	
	Range of Motion	% normal: 100% = 25, 75-99% = 15, 50-74% = 10, 25-49% = 5, 0-24% = 0 or Dorsiflexion-plantarflexion arc: ≥120°=25, 91-119°=15, 61-90°=10, 31-60°=5, 0-30°=0	25	
	Grip Strength	100% = 25, 75-99% = 15, 50-74% = 10, 25-49% = 5, 0-24% = 0	25	
	Total		100	Excellent: 90-100; Good: 80-89; Fair: 65-79; Poor: <65
